# Making drugs from T cells: The quantitative pharmacology of engineered T cell therapeutics

**DOI:** 10.1038/s41540-024-00355-3

**Published:** 2024-03-18

**Authors:** Daniel C. Kirouac, Cole Zmurchok, Denise Morris

**Affiliations:** 1 Notch Therapeutics, Vancouver, BC Canada; 2 The University of British Columbia, School of Biomedical Engineering, Vancouver, BC Canada; 3 Emerald City Pharma Consulting, Blaine, WA USA; 4 Present Address: Metrum Research Group, Tariffville, CT USA

**Keywords:** Cancer, Computer modelling, Pharmacokinetics, Immunology

## Abstract

Engineered T cells have emerged as highly effective treatments for hematological cancers. Hundreds of clinical programs are underway in efforts to expand the efficacy, safety, and applications of this immuno-therapeutic modality. A primary challenge in developing these “living drugs” is the complexity of their pharmacology, as the drug product proliferates, differentiates, traffics between tissues, and evolves through interactions with patient immune systems. Using publicly available clinical data from Chimeric Antigen Receptor (CAR) T cells, we demonstrate how mathematical models can be used to quantify the relationships between product characteristics, patient physiology, pharmacokinetics and clinical outcomes. As scientists work to develop next-generation cell therapy products, mathematical models will be integral for contextualizing data and facilitating the translation of product designs to clinical strategy.

## Introduction

Genetically engineered T cells have proven highly efficacious in treating B cell malignancies, generating durable tumor responses and even cures with a single dose. Six chimeric antigen receptor T cell (CAR-T) therapies have been approved by the FDA since 2017, targeting either CD19 or B Cell Maturation Antigen (BCMA). Efficacy varies by indication and product, but complete response rates typically exceed 50% and extend beyond a year^1^. This is remarkable for treatment-refractory cancers and for patients who have progressed on multiple lines of chemotherapy. These results have galvanized the field, and hundreds of CAR- and TCR-engineered T-cell therapies are now in clinical development for the treatment of a range of cancers and immune disorders^2^. The ability to engineer and deliver targeted cellular immunity offers the potential to tackle diseases with limited treatment options^3^.

Currently, all approved CAR-T products are autologous (derived from patient blood draws), though allogeneic (healthy donor- or stem cell-derived) products are in clinical development. T cells are isolated from a patient’s blood, modified using lentiviral vectors or other synthetic biology approaches, expanded using cytokine cocktails and CD3-stimultion ex vivo, then infused back into patients. While effective, these ‘living drugs’ make unruly therapeutics. The infused T cell compositions actively traffic between tissues, proliferate, differentiate, and interact with patient immune systems in complex and poorly understood ways. If engineered T cells are to fulfill their promise as a groundbreaking therapeutic platform, it is critical to understand their unique pharmacology, and further leverage this understanding to improve product design, treatment regimens, and clinical outcomes.

Developing a novel therapeutic agent from discovery through pre-clinical and clinical stages requires a quantitative understanding of *what the body does to the drug* (pharmacokinetics) and *what the drug does to the body* (pharmacodynamics). This involves the use of mathematical and statistical models to characterize absorption, distribution, routes of elimination, and the relationship between drug exposure and pharmacological activity. Pharmacokinetic-Pharmacodynamic (PKPD) modelling principles were first introduced in the “age of small molecules”^4^. The original mathematical models were highly empirical; they described the pharmacokinetics and exposure-response relationships in patient populations without regard for underlying biological mechanisms. These principles were translated to antibody therapeutics as they emerged in the 1980s, and now to the burgeoning varieties of engineered biotherapeutics^5^. As therapeutic agents have become more complex, the modelling approaches used to characterize their behavior have as well. State-of-the art methodologies now incorporate mechanistically detailed descriptions of ligand-receptor interactions (mechanistic-PKPD)^6^, the physicochemistry and physiology affecting exposure and tissue distribution (physiologically-based PK)^7^, and the cellular biochemistry mediating efficacy and toxicity (systems pharmacology)^8^.

Although model-informed drug development has been widely adopted by both industry and regulatory bodies^9^, integration of these quantitative methods into the nascent CAR-T field has been sporadic. While the underlying biology of T-cell therapies may be complex, we believe the insights from mathematical model-based analyses can facilitate cell therapy research and clinical development.

While there are hundreds of review articles detailing the molecular biology of T cells and advances in chimeric antigen receptor T cell (CAR-T) engineering and clinical data (e.g.^1–3^), there are only a limited number of articles highlighting mathematical approaches which have been employed to describe the pharmacology of CAR-Ts^10–14^. The latter are technical pieces written for pharmacometricians rather than biologists or clinicians, and do not tangibly relate fundamental concepts in quantitative pharmacology (bioavailability, distribution, and clearance) to emerging clinical data, nor how CAR-T product design and patient physiology can modulate key pharmacological parameters. Herein, we take a novel approach to connect these concepts, using simulations of published mathematical models for demonstrative purposes.

We first outline the principles of pharmacology, their application to adoptive T-cell therapy, and the fundamental challenges faced in clinical development of these agents. We then use model simulations to study the effects of cellular heterogeneity and product-intrinsic variance, biodistribution, lymphodepletion response, and allogeneic elimination on CAR-T pharmacokinetics. Finally, we provide perspectives on how data from in vitro functional assays can be integrated with CRISPR-screens and single-cell sequencing to inform clinical development.

## Results & Discussion

### Principles of pharmacology: Pharmacokinetics and Pharmacodynamics

**Fig. 1:**
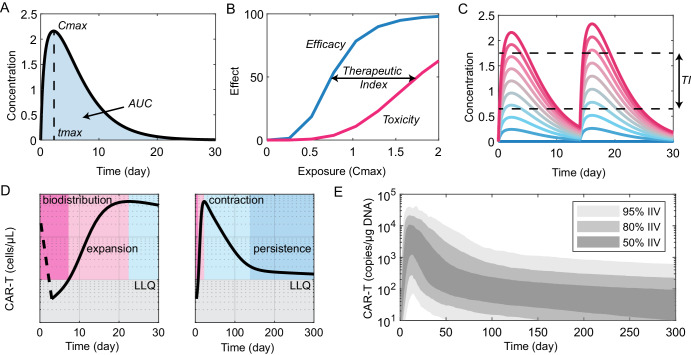
Pharmacology and living drugs. Pharmacokinetic plots (**A**) represent drug concentration over time following administration. *Cmax* (maximal concentration), *tmax*, and *AUC* (concentration-time integral) are used to quantify these curves across populations. Exposure-response analyses (**B**) links drug exposure (e.g., *Cmax* or *AUC*) to drug efficacy and toxicity readouts, typically quantified using Hill-type equations. Separation of the efficacy and toxicity curves is referred to as the therapeutic index (TI). PKPD model simulations (**C**) are used to optimize dosing regimens (dose and schedule) which maximize efficacy while minimizing toxicity (each colored line represents a different dose). CAR-T pharmacokinetics (**D**) can be subdivided into four phases. Immediately following administration is a biodistribution phase, where circulating cell counts rapidly decline, typically to below the limit of quantification. This is followed by an expansion phase, where CAR-Ts proliferate for approximately 2 weeks up to a maximal expansion (*Cmax*), followed by a period of rapid contraction and then persistence (slow clearance). **E** The empirical population PK model of Kymriah simulated for 1000 virtual patients and represented via percentiles of inter-individual variability (IIV).

Pharmacokinetic (PK) curves describe the time-course of drug concentration following administration (Fig. 1A). There are a few key exposure metrics used to quantify these time courses. The maximal concentration reached following administration (*Cmax)*, the time at which this occurs (*tmax)*, and the area under the concentration-time curve (*AUC*). These parameters depend upon the dose, bioavailability of the compound, and physiological mechanisms mediating distribution and elimination. Pharmacodynamics (PD) describes the onset, intensity, and duration of a drug response (efficacious or adverse) and how it relates to the concentration of the drug at the site of action.

Exposure-response (ER) analyses (or pharmacokinetic-pharmacodynamic [PKPD] modeling) are used to relate metrics of drug exposure (e.g., concentration or AUC) to measures of both efficacy and toxicity (Fig. 1B). The variance in exposure necessary to build this relationship can be achieved either from dose-ranging studies and/or by utilizing the observed variability in pharmacokinetic parameters across a patient population. The window between efficacy vs. toxicity is referred to as the therapeutic index and can be quantified by examining the estimated *EC*_*50*_ values. Ideally, one would like to have as wide a therapeutic index as possible. However, what is considered acceptable depends on the indication. For example, a much narrower therapeutic index would be acceptable for a cancer treatment compared to over-the-counter pain relief.

As a drug advances through development, our understanding of the pharmacokinetics, exposure-response relationships, and relevant covariates (patient and disease characteristics) evolves. The end goal of PKPD modelling is typically to design an optimal dosing regimen which maximizes efficacy while minimizing toxicity across the target population (Fig. 1C). So how do these foundational principles apply to CAR-T cell therapies?

### Pharmacology of T cell therapy

The pharmacokinetics of adoptive T cells can be evaluated by blood sampling, either via flow cytometry or PCR-based detection of the CAR transgene. Experience to date with CAR-Ts has shown the behavior, often referred to as *cellular kinetics*, can be segregated into four phases (Fig. 1D).

The first is *biodistribution*. Following infusion, administered T cells rapidly disappear from circulation, and blood concentration will drop by orders of magnitude within a few days^15^. The kinetics of this process are poorly characterized, as the dense time sampling required over the first few hours following administration is rarely performed or reported. Likely the cells actively traffic from circulation into tissues, but the interplay between the cell characteristics, target and anatomic pathology, and the clinical implications remain unknown. Notably, the same phenomenon has been observed for other T cell activating therapies such as IL15^16^ and CD3-bispecific engagers^17^. The biodistribution phase thus appears to be a feature of T cell activation rather than specific to CAR-T dosing.

The subsequent phases are *expansion*, followed by *contraction* and then *persistence*. Cell numbers in the blood rapidly expand for approximately two weeks as the cells encounter antigen and proliferate. After reaching *Cmax*, circulating cell numbers begin to rapidly *contract*. As antigen is cleared, active effector T cells either die or convert to long-term memory T cells^18^. This rapid contraction phase precedes a period of long-term *persistence* or gradual decline, which can last a decade or more^19^. While comprising only a small fraction of circulating T cells (<0.5%), long-term persistent CAR-T cells acquire a distinct phenotype and transcriptional features indicative of antigen stimulation, driven by CD19/BCMA-expression on both healthy and cancerous B cells. Presumably, this population responds to remanent tumor cells as they arise thereby maintaining durable responses^20^.

While the biology underlying these phases may be unclear, empirical mathematical models have been used to quantify the cellular kinetics of CAR-Ts from clinical and pre-clinical studies^10^. The first CAR-T population pharmacokinetic model was developed by Novartis^21^ and included in the Biologics License Application (BLA) for the CD19-targeted CAR-T Kymriah (tisagenlecleucel)^22^. It is empirical in that the model describes the typical shape of the curves using six parameters, without specifying mechanisms mediating the kinetic phase transitions. Both the population average and variance for each parameter are estimated, thereby converting the pharmacokinetic data into a vector of 12 numbers (see Fig. 1E). The equations and parameter estimates then serve as a computational representation by which new data can be benchmarked and are used for this purpose by FDA reviewers^23^.

### Drug development challenges emanating from CAR-T pharmacology

#### Narrow Therapeutic Index

**Fig. 2:**
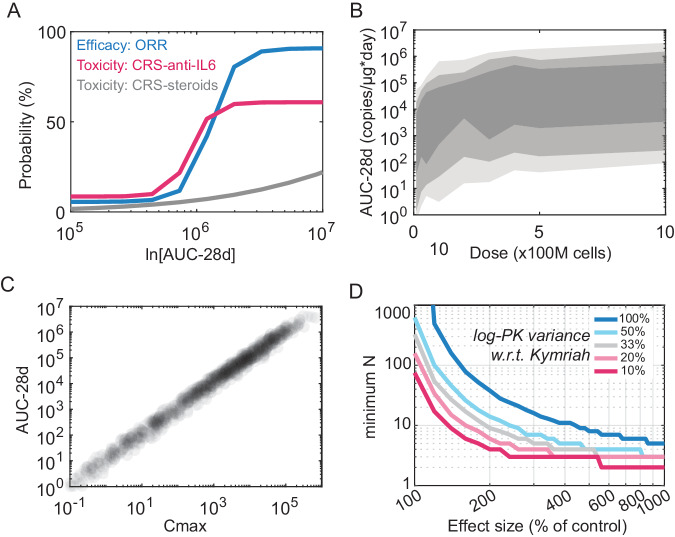
Drug development challenges emanating from CAR-T pharmacology. **A** The exposure-response model reported for Abecma (a BCMA-targeted CAR-T) simulated using reported parameters for efficacy (response rate) and toxicity (Cytokine release syndrome requiring anti-IL6 tocilizumab or steroids) as functions of 28-day *AUC*. **B** Distribution of 28-day *AUC* from Kymriah population simulations over a two-order magnitude dose-range. IIV represented for 95, 80 and 50 percentiles as in Fig. 1E. **C** Correlation between *Cmax* and *AUC* from the Kymriah dose range simulations in **B**. **D** Simulations over a 10-fold range of maximal cell expansion (*Cmax*) effect size vs. a 10-fold range in log-variance were performed. At each point in the grid, minimum sample size (minimum N) requirements for detecting a statistical difference in 28-day log-*AUC* as compared to control were computed (two sample t-test, *p* < 0.05 and 80% power).

Exposure-response analyses for multiple CAR-Ts in different indications reveal that both *AUC* and *Cmax* are predictive of response and toxicity (primarily cytokine release syndrome [CRS]). Patients that fall on the high end of pharmacokinetic exposure are likely to have robust tumor shrinkage, but also experience grade 3/4 CRS with little to no therapeutic index (e.g., the dose vs. response and CRS curves overlap for Abecma, a BCMA-targeted CAR-T approved for the treatment of multiple myeloma; Fig. 2A**)**. This tight correlation points to important biology – the same mechanisms underlying efficacy also mediate toxicity. Activation of circulating T cells and systemic production of inflammatory cytokines is required for tumor clearance, but these processes also cause CRS. Dose fractionation may be an approach to separate CRS from efficacy, as fractionation has been an effective strategy to manage similar toxicities encountered with bispecific T-cell engagers^24^. Preliminary data from a small number of clinical CAR-T trials seem to support this idea^25^.

#### High interpatient variability

Inter-patient variability in exposure (both *Cmax* and *AUC*) is much wider for CAR-Ts than small molecules or biologics. “Highly variable drugs” are defined by the FDA as those for which inter-subject variance (%CV) in exposure is greater than 30%^26^. Highly variable drugs are usually considered an exception and problematic for both developers and regulators. For approved CAR-Ts, PK variance typically spans three orders of magnitude (Fig. 1E), which would terminate a typical drug program. In addition, for small molecules or biologics, drug developers hope to see a clear and consistent relationship (e.g., doubling the dose should correspondingly double the exposure). For CAR-Ts this relationship is often obscured by the high inter-patient variability (Fig. 2B). In other words, inter-patient variance in exposure typically exceeds the range of dose-dependent effect sizes, necessitating many more patients than are typically enrolled in an early clinical study to detect a statistically significant relationship. Exposure is primarily driven by variance in maximal cell expansion (*Cmax*) rather than dose (Fig. 2C). This result is not surprising since CAR-T expansion is antigen-dependent^27,28^ and is corroborated by clinical observations^29^. Given the weak correlation between dose and exposure, CAR-T therapies cannot be dose-optimized as expected for other therapeutic modalities^30^. Thus, exposure-response analyses are useful for retrospective analyses but have little use in prospective decision making.

Insights into the sources of this variance can be gleaned from data as well as theory. In contrast to clinical studies, dose-exposure relationships for CAR Ts are often observed in pre-clinical in vivo studies^31^. These experiments are typically performed with a single batch of starting material and use genetically homogenous mice with uniform tumor xenografts. That is, pre-clinical studies actively minimize variability in both starting material and ‘patient’ populations. Systematic meta-analyses of clinical endpoints has revealed that BCMA-targeted products have less variance and a tighter dose-exposure relationships than CD19-targeted products^32^. These observations indicate that some of the pharmacokinetic variance may be attributable to differences in cancer biology, tumor burden and antigen expression patterns, while the remaining may be attributable to differences in T cell starting material, CAR constructs, or other complex, poorly defined physiological variables.

The wide variability in exposure creates significant challenges for clinical development. Consider the recent FDA guidance on implementation of ‘umbrella trials’ for cell therapy products^33^. Umbrella designs enable the testing of multiple product iterations under a single, multi-arm trial with a single master-protocol and Investigational New Drug (IND) submission. Under this framework, cell therapy developers could test alternate product designs (gene editing strategies, CAR variants, manufacturing protocols, etc.) head-to-head in a clinical setting. However, success is contingent on the ability to make relatively rapid decisions on the clinical benefit of alternative treatment arms, which in turn depends on the treatment effect size relative to the population variance. Observing a statistically significant treatment effect will be challenging for CAR-T products that exhibit large variance. To demonstrate this, we implemented simulations of the Kymriah population pharmacokinetic model as a benchmark CAR-T. Specifically, we used this model to assess how many patients per arm are necessary to detect a statistical difference in tumor response, using simulated exposures (*AUC-28*) as a surrogate (Fig. 2D). With 10 patients included in each arm of the trial and the same PK variance as Kymriah, observing a statistically significant effect of an alternate product design would require a 500% effect size (5-fold increase compared to control). Such large effect sizes seem unlikely under the guise of an umbrella trial. A more reasonable 200% effect size would necessitate 40 patients per treatment arm. However, if the PK variance was substantially reduced, the required number of enrolled patients would be much lower. Considering that pivotal clinical trials costs upward of $40,000 USD per patient^34^, and would be substantially higher for early cell therapy studies. Reducing PK variability is thus a pre-requisite for making multi-arm umbrella trials and iterative clinical development both practically feasible and financially viable for CAR-T therapies.

### Predictive biomarkers of CAR-T product quality and clinical outcomes

Both *Cmax*^35^ and immunophenotype of circulating CAR-Ts following expansion are predictive of patient response. That is, patients with robust and durable tumor responses are more likely to have PK profiles in the top quartile of the distribution (Figs. 1E and 2A), and circulating CAR-Ts comprised of larger proportions of effector rather than exhausted and regulatory T cells^36–38^. While a predictive model of response based on an early clinical readout (e.g., a two-week blood draw) could be informative for subsequent patient care, it would be much more valuable to accurately predict outcomes and modify the treatment regimen before initiation (ideally prior to lymphodepletion). Current release criteria for CAR-T products are CD4/CD8 expression, CAR expression, cell viability and cell number. However, none of these metrics are predictive of clinical outcomes^39–41^ and there are anecdotal reports of CAR-T products which failed release testing yet yielded robust and durable responses^42^. Therefore, quantitative, robust metrics relating pre-infusion product characteristics (e.g., T cell subpopulation phenotyping, cytokine-release assays, transcriptome profiles) to clinical outcomes are needed for both batch release criteria and for process optimization studies.

The frequency of memory vs. exhausted T cell sub-populations has been correlated with response in multiple clinical studies^43,44^. However, the specific markers and flow cytometry gating strategies used to evaluate this are inconsistent and the phenotype-response correlations are generally low. The correlations thus do not robustly translate between clinical studies with differing patient populations and CAR-T manufacturing strategies^45^. In vitro cytokine-release assays have also been shown to correlate with response^46^, albeit with limited study sizes and highly inconsistent metrics. A series of publications have reported transcriptome profiles of pre-infusion CAR-T products matched with clinical outcomes^38,47,48^. In theory, these types of datasets are amenable to unbiased, machine learning approaches to identify predictive biomarkers of response. Yet, they are limited by the “*large P, small N problem*”. The sample numbers (*N* < 30) are too small for unbiased statistical models to extract predictive features from the large number of measurements (*P* ~ 20,000 genes). Intelligent feature engineering strategies are thus required to pre-process the data. Gene set enrichment analyses, for example, have identified transcriptome signatures correlated with response from such studies; signatures for memory, exhausted, and NKT-cells, as well as inflammatory signaling^48^. These are however correlations observed in single studies, rather than predictive models. By combining multiple transcriptome datasets, we were able to train a machine learning classifier using a panel of 28 gene signatures that is highly predictive of response in different indications^45^. Clinical outcomes thus emanate in part from characteristics of the infused cell population, and these attributes, to varying extents, were shared between clinical studies, diseases, and CAR-T products. As publicly available transcriptome data accumulates (e.g.^49^), individual studies can be combined for integrated analysis and refinement of predictive classifiers.

### Using mathematical models to extract product- and patient-intrinsic factors impacting CAR-T pharmacology and clinical outcomes

#### Memory cell proliferative capacity and product-intrinsic variance

**Fig. 3:**
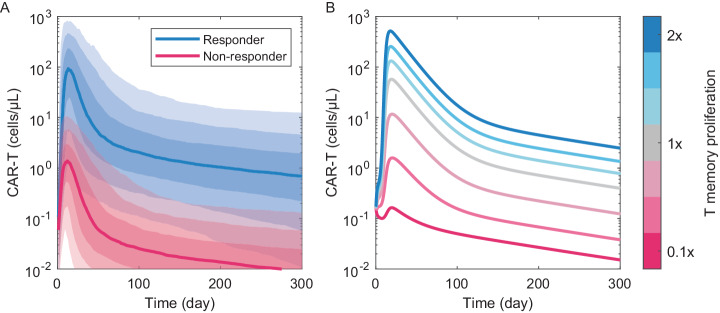
Memory cell proliferation rate can explain pharmacokinetic differences underlying response. **A** The population pharmacokinetic model of Kymriah^21^ simulated with alternate Cmax (fold expansion) parameters reported for responders vs. non-responders in B-ALL^35^. **B** The mechanism-based PKPD model of Kymriah^45^ simulated over a range of memory cell proliferation rates, varying from 0.1 to 2-fold the estimated value.

Despite the extensive literature on mathematical models of T cell-tumor interactions^50^, only a few mechanism-based models have been trained using clinical CAR-T PKPD data^51,52^. Parameterizing such models with data from responders and non-responders can identify explanatory variables underlying response and grounded in established biology. Using data from a study of Kymriah in chronic lymphocytic leukemia (CLL)^44^ and a mechanism-based model of CAR-T cell-tumor antigen interactions, we found the turnover rate of memory cell sub-populations in the CAR-T product to be a key differentiator of response, while memory cell frequency in the infused product was not^45^. This finding was confirmed using single cell transcriptomes from a handful of unrelated clinical studies; CAR-T products resulting in poor clinical response contained memory sub-populations with transcriptional features of functional exhaustion, despite similar immunophenotypes. A subsequent paper reached concordant conclusions i.e. cell-intrinsic functional measures (proliferative capacity and cytotoxic potency) rather than immunophenotypes underly differences in response^53^. Prospective simulations of the model reveal memory cell proliferation rate as a sufficient explanatory variable underlying exposure and response (Fig. 3).

Together, these results suggest that we could improve clinical outcomes if we could design CAR-T products containing memory cells with robust proliferative capacity. However, there are a few hinderances to doing so. First, proliferative capacity may be intrinsic to the patient-derived starting material. That is, autologous T cells may be of variable quality, which in turn determines quality of the resulting CAR-T product. Evidence for this lies in reports that healthy donor-derived T cells make more functionally active CAR-Ts with less batch-to-batch variability^54,55^, and the proliferation rate observed during the ex vivo expansion phase of manufacturing is predictive of clinical response^40^. Differences in T cell quality between disease states may additionally contribute to differences in clinical activity of expanded autologous products. For example, T cells from CLL patients appear more functionally exhausted compared to other lymphomas^56^, and response rates to the derivative CAR-Ts are correspondingly lower. Second, there are no established protocols for consistently generating proliferative memory cells in culture. Although, shorter duration cultures with supportive cytokine cocktails seems to at least preserve memory cell function, improving clinical expansion, persistence, and activity^57,58^. Ultimately, the definition of product-intrinsic vs. patient-intrinsic is somewhat circular for autologous cell therapies. Patients from whom better CAR-T products are derived (enriched with highly proliferative memory cells) may have systemic cytokines or immune microenvironments supportive of such cells. The relative importance of cell- vs. patient-intrinsic variables are thus confounded, as the pharmacology depends on communication between the (patient-derived) product composition and patient physiology.

#### T cell biodistribution effects on CAR-T pharmacology

Although small molecules and biologics distribute throughout tissues following administration, blood is typically a reasonable surrogate of tissue concentration. However, this may not be the case for T cell therapies.

Comparison of infused cell numbers vs. circulating CAR-T cell counts reveals the majority of cells infused are not present in the blood at first measurement (typically taken one to a few days after dosing). To directly compare between studies, we present a simple metric, termed the *Expansion Ratio* (*Cmax* × Blood Volume/Cell dose), and report this for select clinical and preclinical studies (Table 1). The *Expansion Ratio* estimates how many cells appear in circulation at the peak concentration per cell dosed. For preclinical studies this value is less than 1/100 and for clinical trials, expansion ratios >1/10 seem to be a prerequisite for tumor response. Thus, even in responders with a high *Cmax* and multi-log expansion, circulating T cell counts typically just approach input cell numbers.

**Table 1:** Expansion ratios and dependent variables for select clinical and pre-clinical studies

CAR-T	Indication	Cell dose (cells)	Cmax (cell/µL)	Expansion ratio	Reference
CD19 (Cy/Flu)	NHL	1.4 × 10^9^ *^1^	500	2	Turtle^29^
CD19 (Cy)	NHL	1.4 × 10^9^ *^1^	0.2	1/1400	Turtle^29^
CD19 (patient 1)	CLL: Responder	1.1 × 10^9^	20*^2^	1/10	Kalos^15^
CD19 (patient 2)	CLL: Partial Resp.	5.8 × 10^8^	0.2*^2^	1/600	Kalos^15^
CD19 (UCART19)	B-ALL: Responders	10^8^*^3^	10–1000	1/2 - 50	Dupouy^71^
CD19 (UCART19)	B-ALL: Non-responders	10^8^*^3^	<1	< 1/20	Dupouy^71^
CD19-CART	Lymphoma, mix	10^8^*^4^	10–1000	1/3 - 70	Kochenderfer^101^
Kymriah popPK 95%	B-ALL	10^8^*^5^	300*^6^	15	Stein^21^
Kymriah popPK 5%	B-ALL	10^8^*^5^	10*^6^	1/2	Stein^21^
BCMA 1-R2	Preclinical: MM.1 S	10^7^	20	1/250*^7^	Sommer^31^
Kymriah	Preclinical: NALM6	5 × 10^6^	20	1/125*^7^	Stein^102^
iPSC-gdT-CD19	Preclinical: NALM6	10^7^	2	1/2500*^7^	Wallet^103^

*^1^ 2 × 10^7^ cells/kg; assume 70 kg, 5 L blood volume.

*^2^reported as total cells; assume 5 L blood volume.

*^3^weighted average = 10^8^. DL1 (*n* = 6) = 6 × 10^6^, DL2(*n* = 12) = 8 × 10^7^, DL3(*n* = 7) = 2 × 10^8^; no dose-response.

*^4^1–2 × 10^6^ cells/kg, assume 70 kg, 5 L blood volume.

*^5^ median dose 3 × 10^6^ cells/kg for patients ≤ 50 kg, and 10^8^ for patients > 50 kg.

*^6^ scaled counts/ug DNA to cells/µL using data from Kalos^15^.

*^7^ Assume 2 mL total blood volume in mice.

A T cell biodistribution study performed in mice using unmodified, radio-labelled T cells found most of the infused cells rapidly accumulate in the lungs, spleen, liver, kidneys, and lymph nodes^59^. Quantification of tissue vs. blood exposure revealed that for every cell detected in the blood, approximately 1000 are tissue-resident. A similar pattern was observed with CD19-CAR-Ts, with particularly rapid accumulation in lungs within the first day of infusion^60^. It is perhaps not surprising that lung vasculature would be a physical bottleneck, and imaging studies have noted large multi-cellular aggregates of CAR-Ts and B cells trapped in the lung vascular network within 15 minutes after dosing^61^. While human biodistribution data is not available, PBPK modelling predicts lung vasculature as the primary sink, accounting for >99% of administered T cells^62^.

**Fig. 4:**
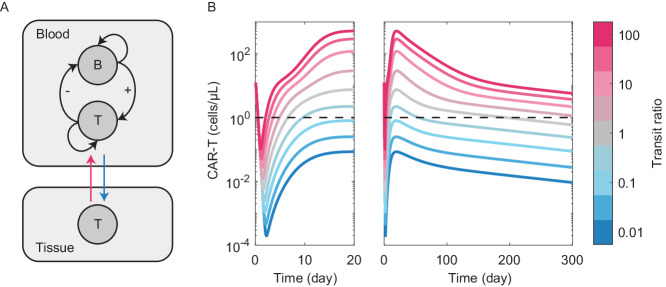
T cell biodistribution may have profound effects on CAR-T pharmacokinetics. **A** Inclusion of a ‘tissue’ compartment, with CAR-T cell distribution between blood and tissue described using two kinetic parameters – tissue extravasation (blue) vs. blood re-circulation (red). For simplicity, we assume CAR-T cells (T) interact with tumor cells (**B**) in the blood, while the tissue compartment acts as a sink. **B** Circulating CAR-T pharmacokinetics, simulated over a four-log range of blood:tissue distribution rates. Dashed line represents a theoretical LLQ.

We explored the theoretical implications of this phenomenon using a minimal adaptation of our mechanistic CAR-T PKPD model^45^. By inclusion of a ‘tissue’ compartment into which administered cells redistribute, PK profiles were simulated over a range of scenarios (Fig. 4). Rapid tissue biodistribution due to extravasation (trafficking of T cells from circulation into surrounding tissues) and the degree of cell ‘stickiness’ (tissue extravasation vs. re-circulation rates) can have a dominant effect on exposure. For example, we can simulate a hypothetically effective CAR-T with robust expansion and *Cmax*, yet create an ineffective therapy that barely breaches the lower limit of quantification (LLQ) in circulation by simply increasing the tissue-stickiness.

Understanding where the administered cells accumulate, how much this varies between patients and populations, and whether these biodistribution patterns contribute to pharmacology and response seem to be important, yet relatively neglected, clinical problems. Whole-body fluorescence or radio-isotope imaging technologies could potentially be implemented to shed light on these unknowns^63^.

Biodistribution is particularly pertinent as clinical programs extended beyond hematological malignancies. With very few exceptions, CAR-T therapy has been ineffective in solid tumors. While reasons for this lackluster efficacy are multi-fold and uncertain, inefficient trafficking to the tumor microenvironment appears to be a primary cause^64^. CAR-T expansion is typically orders of magnitude lower in solid tumor indications, likely a consequence of insufficient antigen exposure and CAR-stimulation. While many cell engineering approaches are underway to enhance solid tumor penetration^65^, routine quantification of T cell migratory patterns in vivo would be an important step towards doing so systematically, and eventually informing predictive PBPK-type distribution models.

#### Lymphodepletion response and resource competition between adoptive and patient T cells

Chemotherapy-based lymphodepletion is required to pre-condition patients, or in other words, ‘make space’ for exogenous T cells prior to CAR-T administration. This is typically achieved via a cyclophosphamide (Cy) and fludarabine (Flu) regimen initiated a week prior to CAR-T infusion^66^. The physiological mechanisms which control circulating T cell counts are not fully understood, but cytokine availability is likely a key contributor. For example, the systemic concentration of the cytokine IL7 spikes in response to lymphodepletion^67^, presumably because the ‘sink’ is removed (circulating lymphocytes). If such homeostatic cytokines are produced at a continuous rate, this would set a maximal carrying capacity. Adoptive T cells would thereby compete for limited resources with patient (host) T cells as the immune system regenerates.

**Fig. 5:**
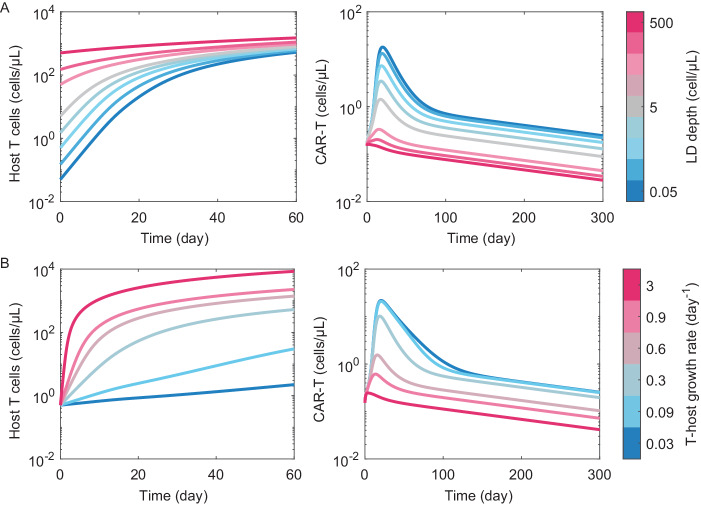
Effects of lympho-depletion and endogenous T cell reconstitution on CAR-T pharmacokinetics. **A** Circulating T cell counts and CAR-T pharmacokinetics following lympho-depletion with varying levels of intensity and fixed reconstitution kinetics. **B** Circulating T cell counts and CAR-T pharmacokinetics following lympho-depletion with a fixed level of intensity with varying T cell reconstitution kinetics.

Clinical trials have shown that the magnitude of lymphodepletion affects the *Cmax* of administered CARTs. More intensive chemotherapy, either via drug combinations (Cy vs. Flu/Cy)^68^ or dosing (30 vs. 60 mg/kg Cy)^67^ enhances the *Cmax* for CD19-CAR-T therapy by approximately an order of magnitude, improving progression-free survival. This phenomenon, endogenous T cells reconstituting alongside the administered CAR-Ts limit growth via competition for resources, can be explored mathematically by the inclusion of host T cells into our mechanistic PKPD model^45^. The depth of lymphodepletion enhances CAR-T exposure by freeing up cytokines and thereby enhancing the proliferation rate of the administered T cells (Fig. 5A). Similarly, the rate at which host T cells reconstitute relative to the administered CAR-T also affects exposure (Fig. 5B). This competitive growth phenomenon has been explored more rigorously using data from the ZUMA-1 trial of Yescarta in diffuse large B cell lymphoma^69^. The authors reach similar conclusions, i.e., competition between exogenous vs. endogenous T cells limits CAR-T expansion, and the variable response to lymphodepletion between patients contributes to variance in CAR-T exposure and patient outcomes.

What does this mean for clinical practice and drug development? The chemotherapy regimens used in current practice were developed empirically, out of experience with hematopoietic stem cell transplantation, rather than from model-optimized design. We imagine there is space for improvement based on ‘standard’ PKPD modelling of Cy/Flu regimens^70^. Newer, targeted lymphodepletion regimens are in development, and have proven beneficial in enhancing activity of CAR-T therapies (e.g., anti-CD52 alemtuzumab^71^). These could likely be further optimized using PKPD modeling approaches. A first step would be routine monitoring and reporting of patient peripheral blood T cell counts alongside CAR-T kinetics. Finally, if cytokine availability limits CAR-T expansion, the cognate signals could be engineered into next-generation products. Indeed, constitutive IL15 receptor signaling constructs have proven effective in improving expansion and activity in pre-clinical studies^31^, essentially a mimetic of lymphodepletion.

#### Allogeneic elimination of administered cells by patient immune systems

All approved CAR-T products, and the majority in development, utilize autologous T cells as starting material. Patient-specific cell manufacturing poses a series of limitations: manufacturing logistics are quite complex, extended ‘vein-to-vein’ wait times negatively affect patient outcomes^72^, the complexity of multi-gene engineering is limited, and variability in starting material leads to products of inconsistent quality. The use of consistent, allogeneic T cells as starting material would potentially alleviate these constraints^73^. However, the main barrier to allogeneic cell therapy is immune cross-reactivity. Administered T cells have the potential to recognize patient antigens and cause life-threatening graft-versus-host disease. Conversely, administered T cells will be recognized as foreign via major histocompatibility complex (MHC) mismatches by a patient’s reconstituting immune system. Thus, in addition to ‘competition for resources’ between exogenous and endogenous T cells, allogeneic CAR-Ts will be subject to additional clearance as a patient’s immunity regenerates. Even for autologous CAR-Ts, there is evidence that humoral and cellular immunity directed against CAR-constructs limits exposure and activity to varying extents^74^.

**Fig. 6:**
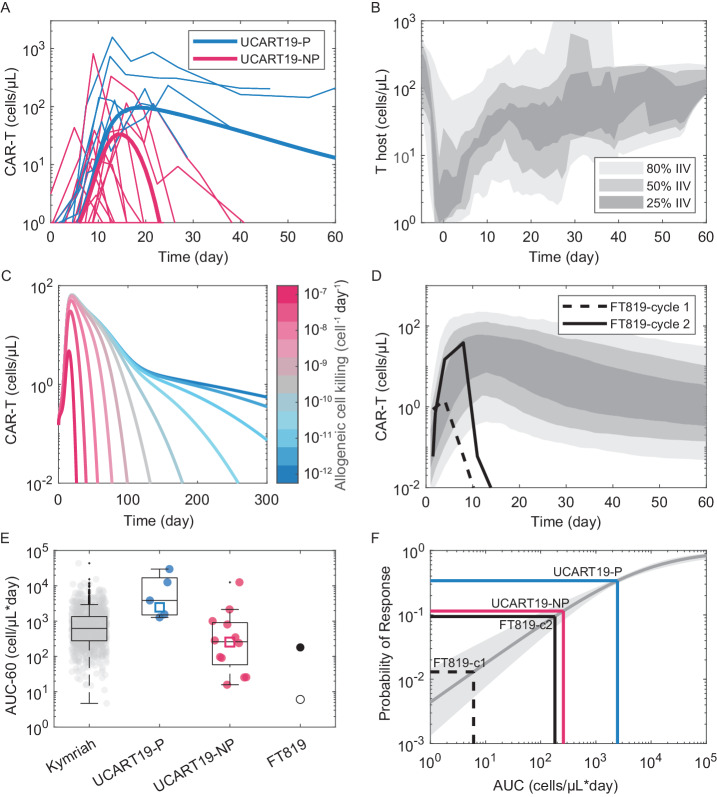
Effects of allogeneic elimination and exemplary clinical data. **A** Reported individual patient pharmacokinetics (*n* = 26) of the allogeneic product UCART19, separated categorically into ‘persisters’ (P) and ‘non-persisters’ (NP)^76^. Raw data overlaid with a modified Kymriah-PKPD model^45^ incorporating allogeneic clearance, tuned to approximately match the population medians for the two patient categories. **B** Host T cell regeneration kinetics following lymphodepletion and UCART19 administration from the same patient cohort. **C** Simulated CAR-T pharmacokinetics over a 5-order of magnitude range of allogeneic elimination rates. **D** Reported individual patient pharmacokinetics (*n* = 1) of the iPSC-derived product FT819, first (cycle 1, 90 M cells) and second dose (cycle 2, 180 M cells)^100^, overlaid with population-pharmacokinetic simulations of Kymriah^21^. **E** Exposure (60-day AUC) calculated for Kymriah, UCART19 and FT819, and simulated for the allogeneic PKPD model tuned to persister (red square) and non-persister (blue square) data. Boxes represent median ± 25 percentiles, and whiskers the min/max or 1.5-times the inter-quartile range from the box outline. **F** UCART19 and FT819 median exposures mapped onto a simulation-based Kymriah exposure-response model in CLL^45^.

The first clinical data reported on donor-derived, allogenic CAR-T therapy was the CALM trial of UCART19 for the treatment of refractory B cell acute lymphoblastic lymphoma (B-ALL). This is a CD19-targeted CAR-T bearing a *TRAC*-knockout, thereby removing donor TCR expression and potential for graft-vs-host disease. The anti-CD52 antibody alemtuzumab was used in addition standard Cy/Flu chemotherapy for enhanced lympho-depletion, but no MHC-gene editing strategies were employed^75^. The product was eliminated on average much faster than the autologous-counterpart Kymriah, with elimination mirroring patient T cell reconstitution (Fig. 6A, B). Moreover, patients could be separated into groups (‘persisters’ and ‘non-persisters’) based on the rate of elimination, a difference explainable solely by differences in allogeneicity (Fig. 6C). Alemtuzumab exposure was identified as a predictor of response status^71^, and a detailed mechanism-based model trained on the data concluded that allogeneic elimination is both the main driver of variance and barrier to durable efficacy^76^.

The use of donor-derived T cells as starting material still poses logistic challenges – notably finding a consistent, rigorously quality controlled source of donor cells for manufacturing. Induced pluripotent stem cells (iPSCs) offer a solution as a potentially limitless source of clonally derived, consistent starting material that could be produced in large batches. Progress has been made in differentiation protocols for generating functionally mature CD8+ T cells from iPSCs^77–79^, and technologies enabling GMP-compatible, scalable batch production are emerging^80,81^. The first clinical data on an iPSC-derived CAR-T product was reported by Fate Therapeutics on FT819, a CD19-targeted CAR-T bearing a *TRAC*-knockout^82^. Efficacy reported in the first data readout was suboptimal, and the single patient PK data showed much faster clearance than autologous or donor-derived CAR-Ts (Fig. 6D). It is not possible to determine from this alone whether the lack of persistence is due to cell-intrinsic deficits, or enhanced elimination by patient immune recognition of the foreign cells. Regardless, mapping computed exposures (60-day AUC; Fig. 6E) to an exposure-response model built on Kymriah in CLL reveals that clearance fully accounts for the deficit in clinical activity (Fig. 6F). A primary challenge for the next generation of iPSC-CAR-T development is thus to first identify the mechanisms responsible for shortened persistence and then implement cell engineering strategies to overcome these deficits. This is a tall order, most likely requiring a combination of improved differentiation protocols, engineered signaling constructs which enhance memory cell generation^83^ plus MHC-editing strategies for immune-evasion^84^.

So far, we have shown the utility of mathematical models in characterizing the clinical pharmacology of engineered T cells. Specifically, we have highlighted the drug development challenges unique to this field, including product heterogeneity, tissue trafficking and biodistribution, lymphodepletion response and allogeneic elimination. These factors can significantly impact pharmacokinetics and efficacy, and model simulations may be employed to inform clinical development strategies. A grand challenge over the coming years will be to integrate clinical insights with advanced research technologies, and to rationally design next-generation products with improved therapeutic profiles. We believe biology-based, quantitative systems pharmacology models will be pivotal in addressing this challenge.

### Translational modelling and next-generation T cell therapies

Mathematical models are essential for product development and in silico prototyping in many industries. While human physiology is too complex and uncharted to fully recapitulate in a computer simulation, model-informed drug development (MIDD) still plays a vital role in decision-making across biopharma^85,86^. The models employed can be aligned on a spectrum from empirical to mechanistic. Empirical models (based purely on data and statistics, encompassing machine-learning to classic exposure-response equations) are useful for quantifying system behavior, identifying important variables, and specifying input-output relationships. However, they are constrained to interpolation within the bounds of the training data. As such, we cannot simulate the effect of dose schedule, tumor burden, T cell composition, lymphodepletion response, etc. if such variables were not included in model training. By incorporating established knowledge and biological hypotheses, mechanism-based models have the advantage of exploring ‘what if’ scenarios in silico^14^.

The sparse implementation of mathematical modelling in the cell therapy space can be attributed to limited data availability. While hundreds of CAR-T clinical trials have been conducted, the patient-level data for the vast majority remains locked. Population-averaged data, when published, is useful but obscures individual variability essential to deepening our understanding of this therapeutic class. Numerous legal, intellectual property and business factors play a role in the decision to withhold clinical data from public release. As an alternative, mathematical models are an efficient means of both encoding clinical results and sharing insights. Stein et al.^21^, for example, did not release the underlying clinical data for Kymriah, but the published mathematical model serves as a computational representation, amenable to subsequent reuse, modification, and benchmarking (as we have demonstrated). More broadly, executable computer code is a more consistent, formalized platform for sharing scientific findings than text and summary figures^87^.

Another primary challenge in model-informed development of T-cell therapies is the inadequacy of pre-clinical models. Therapeutic development relies upon the use of rapid and high-throughput in vitro and in vivo surrogates of clinical activity^88^. For therapeutic T cells, the gold standard in vitro functional surrogate is the serial killing assay (repeated target cell lysis)^89^. While it is routinely stated that pre-clinical models are ‘not predictive’, there exists, to our knowledge, no systematic studies qualifying this statement. We were unable to identify studies comparing in vitro or in vivo pre-clinical functional readouts to clinical data for CAR-T therapies. Moreover, the raw data readouts from such assays (cell counts) are highly dependent upon specifics of the assay design (i.e. time, Effector:Target ratio, cytokine supplements, etc.), which likely contributes to this pessimism. However, model parameters such as kinetic rate constants inferred from such data are more invariant to specifics of experimental design^90^ and thus facilitate clinical predictions.

**Fig. 7:**
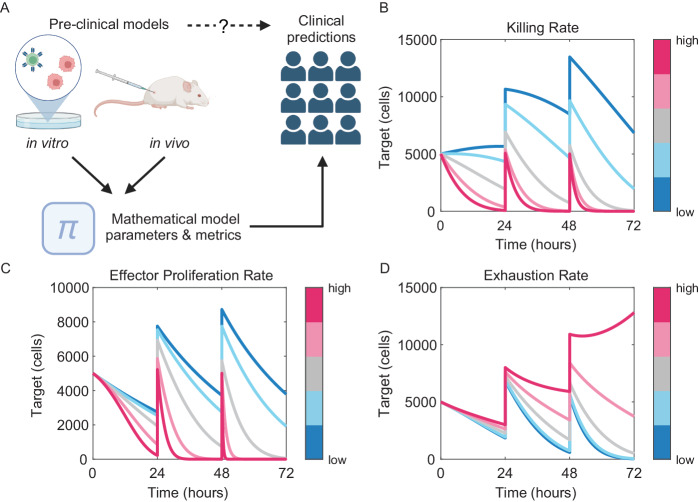
Quantification of in vitro functional assays for translational systems pharmacology modelling. **A** CAR-T cell proliferation and tumor cell dynamics measured in in vitro and in vivo functional assays functional data can yield insights into fundamental biology and facilitate clinical predictions when quantified with systems pharmacology models (Created with BioRender.com). Model simulations of CAR-T cell and tumor cell interactions in a 24-hour serial restimulation assay reveal the complex, non-linear effects of drug product characteristics such as CAR-T cytotoxicity (**B**), proliferation (**C**), or exhaustion (**D**) on tumor cell survival.

Simulations of a modified version of the ‘CARRGO’ model^91^ (itself a derivative of the classic “predator-prey” model) reveal that the shape of tumor dynamic curves from serial re-stimulation assays can be quite complex, and interpretation non-intuitive (Fig. 7). However, fundamental features of T cell function (proliferation rate, cytotoxic activity, and exhaustion) yield distinct features that can be inferred using careful experimental design coupled with mathematical analyses. The effect of gene edits or other cell engineering strategies on such kinetic rates could be mapped onto PKPD models for clinical predictions. One such multi-scale translational model was developed to understand the dose-response properties of the BCMA-targeted CAR-T Abecma (bb2121), connecting in vitro, in vivo and clinical data^92^. Though not used (to our knowledge) for prospective product design or lead selection, this model proves the technical feasibility of doing so.

A critical yet unresolved issue in the T cell therapy space is the fact that what makes an optimal phenotype for adoptive therapy remains elusive. Without robust critical quality attributes (CQAs) linking molecular features to function, it is not possible to rationally specify design or release criteria. The potential design space is so large (CAR/TCR designs, gene edits, synthetic biology-based regulatory switches, cell sources and expansion protocols) and the mechanisms linking molecular perturbations to function so complex and non-linear, classic one-at-a-time hypothesis testing is not a practically feasible approach to map the design space. High-throughput CRISPR-library-based screens coupled with single-cell transcriptome-based phenotyping and functional assays could however be utilized. These have proven effective in the design of novel CAR signaling constructs^93,94^ and the identification of functional gene knock-ins^95^. Coupling these datasets with biologically-informed mathematical models (i.e., signaling and transcriptional regulatory networks^96,97^) could be instrumental in both specifying CQAs, and predictively linking these attributes to cell culture inputs, a step towards Quality By Design for cell therapy manufacturing^98^.

Cell-based therapies are inherently more complex than other treatment modalities. A common refrain is “it’s too complicated to model” and the requisite simplifying assumptions ignore the nuances of biology. Instead, we see this as a strength. Model development (the careful specification of important variables along with the hypothetical or known mechanisms connecting them) and model calibration (the degree of certainty in the model and consistency with data) are valuable pressure tests. The resulting mathematical models are then quantitative representations of our mental constructs, and simulations are simply the consequences of our assumptions and assertations.

There is flurry of research and development work in the T cell therapy space. Akin to Plato’s Allegory of the Cave^99^, clinical observations and experimental data represent but flickering shadows cast from a deeper and more complex reality. Mathematical models enable one to synthesize those potential realities, test which are most consistent with the “flickering shadows” and explore the consequences of such inferences. If we are to translate the plethora of novel cell designs and manufacturing protocols into the next generation of clinical products, mathematical models will be essential to interpret the shadows, and assemble our perceptions into coherent theory.

## Methods

### PKPD model simulations

A standard two-compartment pharmacokinetic model with linear absorption was simulated for illustrative purposes. Efficacy and toxicity were linked via direct response to serum concentration via Hill equations, and simulations executed over a dose range of 2 through 18 (arbitrary units). Equations were coded in MATLAB’s SimBiology toolbox, and all simulations were executed using MATLAB R2022b.

### Empirical CAR-T pharmacokinetic model simulations

Model equations and baseline parameters describing population pharmacokinetics of Kymriah in B-ALL were taken from Stein et al.^21^. Responder vs. Non-responder simulations were executed by encoding differences in Cmax/fold expansion as reported by Liu et al.^35^ for the same clinical study (R vs. NR Cmax equivalent to 10^5^ vs. 10^3^ counts/µg DNA), and statistics calculated from 1000 simulations (virtual patients). Dose response simulations were executed by scanning initial CAR-T amounts (*C*_*0*_) across a 50-fold scaled range with a median dose of 10^8^ cells representing a dose ranging study of 10^7^ to 5 × 10^8^ cells, approximately equivalent to that reported in the original BLA^22^. Grid simulations of CAR-T cell expansion effect size vs. population variance were executed by simulating the model (*n* = 10,000 virtual patients) with the maximum cell expansion ‘Cmax’ parameter scaled from 1- to 10-fold the reported mean value (24000 copies/µg), and the log-variance (‘OMEGA’) scaled from 1 to 0.1-fold the reported range across all six model parameters. AUC_28d_ was calculated based on a daily sampling schedule, and minimum sample sizes were estimated at each point in the grid by comparison to the control AUC_28_ using a two-sided t-test with a power of 80% and significance of 0.05, using MATLAB’s *sampsizepwr* function.

Translation between counts/µg DNA to cells/µL was estimated using CD19-CAR-T data reported by Kallos et al.^15^: we estimate 1 count/µg DNA ≈ 0.05 cells/µL.

### Mechanistic CAR-T pharmacokinetic simulations

To simulate the effect of memory cell proliferation rate on CAR-T pharmacokinetics, the mechanism-based CAR-T PKPD model published by Kirouac et al.^45^ was executed with the *µ*_*M*_ parameter varied from 0.1 to 2-times the reported estimate, using a single parameter vector (CR patient #1).

To simulate the effect of T cell distribution into tissues, the published model structure was edited in SimBiology model builder. A second ‘tissue’ compartment was created, witch two parameters quantifying T cell distributing from blood to tissue (rate *k*_*12*_) and tissue to blood (rate *k*_*21*_) based on mass action kinetics. The k_21_ parameter was multiplied by an autonomous (time-dependent) term to simulate a time delay (*TD*) post-infusion as:

1 $${TD}={{time}}^{k}/\left({{{TD}}_{50}}^{k}+{{time}}^{k}\right),$$

with *TD*_*50*_ = 12 days and *k* = 4 for switch-type behavior. With *k*_*12*_ (tissue distribution) fixed at 6 day^−1^, simulations were executed with the blood re-distribution rate (*k*_*21*_) varied over 4 orders of magnitude.

To simulate the effect of lymphodepletion and exogenous vs. endogenous T cell competition, host T cell (*Th*) reconstitution was described using a saturating growth rate ordinary differential equation:

2 $$\frac{{dTh}}{{dt}}={\mu }_{h}\bullet {T}_{K}/\left({T}_{K}+{T}_{t}\right)\cdot {T}_{h},$$

were *T*_*t*_ = *Th* + *T*_*CART*_ (total circulating T cells), ${\mu }_{h}$ is the growth rate of host T cells and *T*_*K*_ the carrying capacity, set at 50 cell/µL such that steady state circulating T cell counts saturate at approximately 10^3^ cell/µL. The CAR-T proliferation rates were all multiplied by the saturation function (${T}_{K}/\left({T}_{K}+{T}_{t}\right)$) such that proliferation is equivalently limited by total circulating T cell counts.

Simulations were executed by varying the initial number of host T cells post-lymphodepletion over 3-orders of magnitude (from 0.05 to 500 cells/µL) while keeping the host T cell growth rate fixed at 0.35 day^−1^, or fixing the post-lymphodepletion T cell count at 0.5 cell/µL while varying the growth rate over 2-orders of magnitude from 0.03 to 3 day^−1^.

To simulate the effect of allogeneic clearance, each equation in the above model was edited to include an additional term modelling direct host T cell CAR-T killing via mass-action kinetics:

3 $$\frac{d{T}_{{CART}}}{{dt}}=\ldots -{k}_{{kill}}\bullet {T}_{{CART}}\bullet {T}_{h}.$$

Simulations were executed by fixing the initial host T cell count (*T*_*h*_) post-lympho-depletion at 0.5 cell/µL and growth rate (${\mu }_{h}$) at 0.35 day^−1^ and varying the allogeneic cell killing rate (*k*_*kill*_) from 10^−12^ to 10^−7^ cell^−1^day^−1^. Persisters vs. non-persisters were simulated by setting the allogeneic cell killing rate (*k*_*kill*_) at 0 and 7 × 10^−9^ cell^−1^day^−1^, respectively.

### UCART19 and FT819 data

CAR-T pharmacokinetic data was digitized using Graph Grabber v2 (Quintessa). For UCART19, individual patient data was extracted from the supplemental figures in Derippe et al.^76^, and FT819 using a figure from an ASH 2022 poster on interim FT-819-101 trial data^100^.

### Simulations of serial restimulation assays

We adapted the CARRGO predator-prey model of CAR-T cell (*E*) and tumor (*T*) cell interactions^91^ to include CAR-T cell exhaustion upon interaction with Tumor cells (rate constant $\nu$):

4 $$\frac{{dE}}{{dt}}=\gamma E-\theta E-\nu {ET},$$

5 $$\frac{{dT}}{{dt}}=\rho T\left(1-\beta T\right)-\kappa {ET},$$

and simulated 3, 24-hour restimulations at 1:1 E:T where 5000 tumor cells were added at the end of each stimulation. Baseline model parameters were $\rho =\gamma =\log (2)/24$ hour^−1^, $\beta ={10}^{-5}$ cell^−1^, $\kappa ={10}^{-5}$ hour^−1^ cell^−1^, $\theta ={10}^{-3}$ hour^−1^, and $\nu ={10}^{-6}$, with $E\left(0\right)=T\left(0\right)=5000$ cells. We simulated the model over a range of parameter sweeps by fold-changing each model parameter multiplicatively one-at-a-time with the following fold changes: 1/3, 1/2, 1, 2, 3 for cytotoxicity ($\kappa$) and proliferation ($\gamma$), and 1/2, 1, 3, 5, 7 for exhaustion ($\nu$).

### Reporting summary

Further information on research design is available in the Nature Research Reporting Summary linked to this article.

### Supplementary information

Reporting Summary

## Data Availability

All data and models were extracted from prior publications.
